# Saline Arterial Injections in Collapse

**Published:** 1882-05

**Authors:** 


### SALINE ARTERIAL INJECTIONS IN COLLAPSE.

It has long been known that exsanguinated animals can often be revived by intra-arterial injections of saline solutions. This fact has not, however, until recently, received a practical application in therapeutics. Dr. J. J. Bischoff has been perhaps the first (*Centralblatt für Gynäkologie*, November 12, 1881) to endeavor to resuscitate a patient in collapse resulting from post-partum hemorrhage, by this means. After the child's birth a considerable quantity of blood was lost by the patient, which loss was still further increased by the delivery of the placenta. The hemorrhage ceased, but the patient collapsed, despite the use of stimulants. An aqueous solution of common salt (six parts to the thousand), rendered slightly alkaline by a little lye, was injected into the radial artery. The patient exhibited none of the oppressive symptoms common to transfusion operations, but rallied rapidly, and fully recovered. The reason assigned by Dr. Bischoff for choosing an artery in lieu of a vein is that in great anæmia the superficial veins are with difficulty found. The procedure would seem capable of further extension.

Venous injections of warm water in cholera have at times been attended with remarkable, though temporary results. The saline injections would seem likely to have similar results of a much more permanent character. —*Chicago Medical Review.*

